# Characterization of *Spodoptera littoralis* (Lepidoptera: Noctuidae) resistance to indoxacarb: inheritance mode, realized heritability, and fitness costs

**DOI:** 10.1093/jee/toae024

**Published:** 2024-02-20

**Authors:** El-Sayed M S Mokbel, Moataz A M Moustafa, Nawal Abdulaziz Alfuhaid, Eman A Fouad

**Affiliations:** Department of Standard Rearing, Central Agricultural Pesticides Laboratory, Agricultural Research Center, 12618 Giza, Egypt; Department of Economic Entomology and Pesticides, Faculty of Agriculture, Cairo University, 12613 Giza, Egypt; Department of Biology, College of Science and Humanities, Prince Sattam Bin Abdulziz University, Al-Kharj 11942, Saudi Arabia; Department of Bioassay, Central Agricultural Pesticides Laboratory, Agricultural Research Center, 12618 Giza, Egypt

**Keywords:** *S. littoralis*, indoxacarb, inheritance mode, realized heritability, fitness cost

## Abstract

*Spodoptera littoralis* (Boisd.) (Lepidoptera: Noctuidae) is a major economic pest attacking a variety of crops in Egypt and other Mediterranean countries. *S. littoralis* has developed resistance to both traditional and novel insecticides. The current study investigated *S. littoralis* resistance to indoxacarb regarding inheritance mode, realized heritability (*h*^2^), and fitness costs. An indoxacarb-resistant strain (Indoxa-SEL) was obtained by selecting a field strain with indoxacarb. Indoxa-SEL strain outperformed the susceptible one (Indoxa-S) by 29.77-fold after 16 consecutive generations of selection. Based on the LC_50_ values of the progenies of reciprocal crosses F1 (R♂ × S♀) and F1ʹ (R♀ × S♂), *S. littoralis* resistance to indoxacarb was found to be autosomal and partially recessive. Chi-square tests for goodness-of-fit between observed and expected mortalities of self-bred F_1_ and resistant strain reciprocal crosses revealed that the resistance was controlled by multiple genes. The resistant strain had a relative fitness of 0.80, with significantly increased total preovipositional period of females, egg, larvae, pupae, preadult, adult, and total longevity period. The estimated realized heritability value in the Indoxa-SEL strain was 0.21. The current study will contribute to sustaining indoxacarb efficacy and designing effective resistance management programs against *S. littoralis*.

## Graphical Abstract

**Graphical Abstract:**
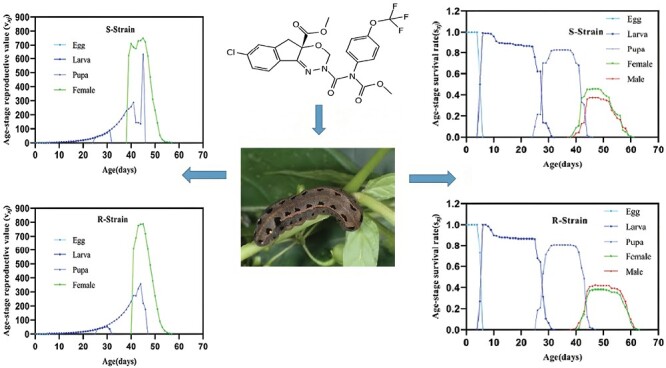

## Introduction

The cotton leafworm, *Spodoptera littoralis*, is a highly destructive polyphagous pest (Mokbel and Huesien 2020). It is distributed throughout the tropical and subtropical regions (Brown and Dewhurst 1975). It causes considerable damage to several economic field crops including cotton, eggplant, tomato, pepper, cowpea, bean, alfalfa, lettuce, spinach, strawberry, cabbage, peanuts, maize, soybeans, and ornamental crops (El-Sheikh et al. 2018). Therefore, more than 33 insecticide formulations, belonging to various groups, have been used in Egypt to control this pest (Egyptian Agricultural Pesticides Committee 2020). Unfortunately, *S. littoralis* has developed resistance to most of these insecticides, creating an urgent need for developing more effective and selective alternative insecticides.

Indoxacarb, an oxadiazine insecticide, is a sodium channel blocker proinsecticide that is bioactivated to the active *N*-decarbomethoxylated metabolite by an esterase’ or amidase’ type of enzyme(s). The active metabolite blocks sodium channels more effectively than indoxacarb, preventing sodium influx into neurons, and thus resulting in insect paralysis and death (Wing et al. 1998, 2010). Indoxacarb is widely used against lepidopteran species (Moustafa et al. 2021, 2023). As a result of this extensive use, resistance to indoxacarb has been developed and reported in the armyworm, *Spodoptera litura* Fabricius (Lepidoptera: Noctuidae) (Shi et al. 2022), and the beet armyworm, *Spodoptera exigua* Hubner (Lepidoptera: Noctuidae) (Gao et al. 2014).

In this respect, genetic studies are of great importance, as they provide findings that can help delay resistance development and preserve insecticide efficiency (Roush and McKenzie 1987, Sayyed et al. 2005). Previously, genetic resistance of indoxacarb has been studied in several insects, including the cotton bollworm, *Helicoverpa armigera* Hübner (Lepidoptera: Noctuidae) (Ghodki et al. 2009), the armyworm, *S. litura* (Sayyed et al. 2008), and the diamondback moth, *Plutella xylostella* L. (Lepidoptera: Plutellidae) (Sayyed and Wright 2006, Nehare et al. 2010). To our knowledge, little research, if any, has been done on the genetics of indoxacarb resistance to *S. littoralis*.

Resistance risk assessment is an effective tool to avoid or delay insecticide resistance. It is crucial for overcoming resistance evolution and insecticide sustainable use (Gao et al. 2014). Insecticide resistance can be predicted through short laboratory selection experiments and realized heritability calculation (Sun et al. 2022). Generally, resistant individuals are rare before insecticide application. The resistant individuals usually exhibit a delay in population growth, fecundity decrease, and longevity shortness (Shen et al. 2017, Tieu et al. 2017, Liao et al. 2019). In addition, several factors affect the resistance resurgence including environmental, biological, and genetic factors of the tested population (Keiding 1986).

The current study aimed to evaluate the inheritance and fitness costs of *S. littoralis* resistance to indoxacarb and to calculate the realized heritability value to assess indoxacarb resistance risk. The obtained data will contribute effectively to designing an effective resistance management program and predicting indoxacarb resistance under various conditions.

## Materials and Methods

### Insects and Insecticide

In this study, *S. littoralis* colony was originally obtained from El-Fayoum (Fs) Governorate, Egypt (Fouad et al. 2022). The insect was reared on insecticide-free Castor bean leaves at 27 ± 1 °C, 70–80% relative humidity (RH), and a 16:8 light/dark (LD) cycle. Two different strains were used in this study. The first one has been maintained at the Standard Rearing Department, Central Agricultural Pesticides Laboratory, Agricultural Research Center, Giza, Egypt since 2018 without any selection pressure and was designated as the susceptible strain (Indoxa-S). The second strain has continuously been selected with indoxacarb for 15 generations under the laboratory conditions and was designated as the indoxacarb resistant strain (Indoxa-SEL). Indoxacarb (Avaunt 15% EC, DuPont) was used in the experiments.

### Bioassays

Concentration-mortality response of indoxacarb against fourth instar larvae of *S. littoralis* was evaluated with the leaf-dip method (Moustafa et al. 2021). Castor bean leaves were dipped in 7 different concentrations of indoxacarb for 30 s, allowed to dry at room temperature, and then were placed in glass jars (15 cm high and 10 cm diameter). Five replicates, each of 10 fourth instar larvae, were prepared for each concentration. The larvae fed on the treated leaves for only 24 h before switching to untreated fresh leaves. Mortality was recorded after 96 h.

#### Realized heritability (h^2^) estimation

The realized heritability (*h*^2^) values of indoxacarb resistance were determined according to the formula of Tabashnik (1992) using the equation $h^{2}=R/S$, where *R* indicates response to selection and is calculated as follows: $R=(log(\;f\;inal\;LC_{50})-log(initial\;LC_{50})/n$, where the final LC_50_ is the LC_50_ of survivors after a number of selected generations, and the initial LC_50_ is the LC_50_ of the parental generation before selection. *S* indicates selection differential (*S*) and is calculated as follows: S=iσp, where *i* is the selection intensity estimated with the formula: $i=1.583-0.0193336p+0.0000428p^{2}+3.65194/p$, where *p* is the average percent survival of the insecticide-selected strain and *σp* is the phenotypic standard deviation and is calculated as: σp=Mean slope ^−1^σp=. The mean slope is the average of the slopes of the respective generations. According to LC_50_ values, the number of generations (*G*) required for a 10-fold resistance was estimated as follows: $G=R^{-1}={\left(h^{2}S\right)}^{-1}$

### Genetic Crosses

To determine the genetic basis of *S. littoralis* resistance indoxacarb, pupae of both susceptible strain (Indoxa-S) and indoxacarb-resistant strain (Indoxa-SEL) were identified as males and females. The males and females of both strains were separated in glass jars to ensure virginity for further use in genetic crosses. Fifty males and females underwent reciprocal crosses between the susceptible strain (Indoxa-S) and the indoxacarb-resistant strain (Indoxa-SEL) to establish 2 F_1_ lines: F_1_RS (R♂ × S♀) and F_1_ʹ SR (R♀ × S♂). Similarly, the backcross F_2_ lines BC_1_ (F_1_♂ × R♀), and BC_2_ (F_1_♀ × R♂) were obtained from the reciprocal progenies of F_1_ crosses with the parental (Indoxa-SEL).

#### Degree of dominance

The degree of dominance (*D*) of the Indoxa-SEL strain of *S. littoralis* was determined using the following formula (Bourguet and Raymond 1998):


$$\begin{array}{l} D_{LC}=Log\;LC_{50}F1\text{-}Log\;LC_{50}\;SS/Log\;LC_{50}RR\text{-}LogLC_{50}SS \end{array}$$

The degree of dominance ranges from 0 (completely recessive) to 1 (completely dominant).

## Effective dominance

The effective dominance (*D*_*ML*_) values were calculated using the following formula (Bourguet et al. 2000):


$$\begin{array}{l} D_{ML}=(MT_{RS}-MT_{SS})/(MT_{RR}-MT_{SS}) \end{array}$$

where MT_RR_, MT_SS_, and MT_RS_ are the mortality levels of Indoxa-SEL, Indoxa-S, and reciprocal crosses, respectively, in different concentrations of indoxacarb. *D*_*ML*_ values range from 0 to 1, where 0 is completely recessive and 1 is the complete dominant nature of resistance. The *D*_*ML*_ values of 0.1–0.5 and 0.6–0.9, show partially recessive and partially dominant indoxacarb resistance patterns, respectively.

## Number of gene(s) conferring resistance to indoxacarb

To assess resistance through monogenic or multiple-gene approach, the goodness-of-fit between the observed and expected concentration response in F_2_ backcrosses was achieved, and the Chi-square (χ^2^) test was used (significant χ^2^ means multiple-gene resistance) (Liao et al. 2019) as follows:


$$\begin{array}{l} Chi\;square\;(\chi^{2})={\left(F-pn\right)}^{2}/pqn \end{array}$$

where *F* is the observed mortality in the tested F2 backcrosses at a particular concentration, *p* represents the expected mortality, *n* is the number of individuals exposed to a particular concentration, and *q* is calculated as (1 − *p*). If the Chi square values between expected and observed were significantly different, then resistance is controlled by multiple-genes, and vice versa (He et al. 2009, Feng et al. 2018).

### Fitness Comparison

Life table analysis were constructed for susceptible (Indoxa-S), and resistant (Indoxa-SEL) populations throughout the age–stage, two-sex life-table approach (Chi and Liu 1985). For each strain, about 50 adults were collected and placed in glass jars covered with Muslin cloth. The glass jars were supplied with 10% sucrose solution for adult nutrition. Furthermore, paper strips were hung in these jars for oviposition. Eggs were gathered daily and transferred to new jars. After hatching, about 100 neonate larvae were randomly transferred individually to plastic cups with fresh Castor bean leaves. The larvae were observed and recorded daily. Castor bean leaves were replaced with fresh ones every 2–3 days until adulthood. Unmated male and female adults were simultaneously paired in small glass jars (0.5 L) containing small balls of cotton immersed in 10% sucrose solution, and paper strips for oviposition and covered with a cloth top. The fecundity and mortality of each pair were recorded until the death of all individuals in each strain. The population growth parameters and developmental times of the different stages were recorded daily. The life table was established according to Chi (1988). The life-table experiment was maintained at 25 ± 1 · C and 65–70% RH under a 14:10 h L:D cycle.

### Statistical Analysis

Concentration-mortality percent were corrected according to Abbott (1925), then subjected to probit analysis (Finny 1971) using the Probit-MSChart computer program (Chi 2023a). Row data of life table experiments were subjected to the TWOSEX-MSChart computer program (Chi 2023b), which based on the age-stage two-sex life table theory (Chi and Liu 1985). Standard error and significant differences for life table and population growth parameters were estimated using 100,000 bootstrap replicates in TWOSEX-MSChart program. The population parameters of the Indoxa-S and Indoxa-SEL strains were compared based on the confidence interval (CI) (if the CI includes 0, there was no difference) of the differences (Liao et al. 2019). Graphs of age-stage specific survival rate (*s*_*xj*_), age-stage reproductive value (*v*_*xj*_), age- specific survival rate (*l*_*x*_), age-specific fecundity (*m*_*x*_), and age-specific maternity *(l*_*x*_*m*_*x*_), and age-stage life expectancy (*e*_*xj*_) were conducted by GraphPad Prism 9. The relative fitness (Rf) of the resistant strain was calculated as


$$\begin{array}{l} Rf\;=R_{0}\left(Indoxa\;SEL\right)/R_{0}\left(Indoxa\;S\right) \end{array}$$

## Results

### Indoxacarb Resistance Selection

A field population of *S. littoralis* was continuously selected with indoxacarb for 16 generations in the laboratory (Table 1). Before laboratory selection, the LC_50_ of indoxacarb was 0.0395 mgL^−1^ for the parent generation (G_0_). The results showed that LC_50_ increased to 0.5729 mgL^−1^ for G_7_ generation. From G_7_ to G_15_, the resistance developed slowly and LC_50_ increased to 0.8516 mgL^−1^. Overall, the consecutive selection with indoxacarb for 16 generations with LC_50_ increased the resistance ratio (RR) to 21.56-fold.

**Table 1.** Slope and LC_50_ values for G_0_, G_7_, and G_15_ used in realized heritability(*h*^2^) calculation

Generation	LC_50_ (ppm)	95% CL	Fit of probit				
			Slope ± SE	χ^2^	df	*P*	RR
G_0_	0.039	0.027–0.059	0.84 ± 0.07	0.176	2	0.916	1.00
G_7_	0.573	0.447–0.718	2.69 ± 0.44	0.954	2	0.621	14.5
G_15_	0.852	0.625–1.205	2.09 ± 0.40	1.004	2	0.605	21.56

CL, the 95% confidence limits of LC_50_.

SE, standard error of the mean value.

χ^2^, Chi square value.

df, degree of freedom.

*P*, the probability value.

RR, resistance ratio for a certain generation and calculated as: LC_50_ for certain generation/ LC_50_for G_0_.

SE, standard error of the mean value.

### Resistance Inheritance Characteristics

The LC_50_ values of indoxacarb for the reciprocal crosses, F_1_ (R♂ × S♀) and F_1_ʹ (R♀ × S♂) showed no significant differences based on the overlapping of 95% (CLs) of the LC_50_ values. The LC_50_ values in F_1_ (R♂ × S♀) and F_1_ʹ (R♀ × S♂) ranged from 0.155 to 0.139 mgL^−1^. The LC_50_ value of the pooled population in F_1_ was 0.146 mgL^−1^ (Table 2).

**Table 2.** Responses of parental, reciprocal crosses, and backcross populations of *Spodoptera littoralis* to indoxacarb

Population	LC_50_ (ppm)	95% CL	The fit of the probit line				RR (95%CL)	Log (LC_50_)	D_LC_
			Slope ± SE	χ2	df	*P*			
Lab-Pop (S)	0.031	0.004–0.177	0.87 ± 0.20	7.241	3	0.064	1.00	−1.508	
Indox-SEL (R)	0.923	0.777–1.114	2.41 ± 0.42	0.208	2	0.901	29.77	−0.034	
F_1_ (R♂ × S♀)	0.155	0.084– 0.297	1.46 ± 0.37	0.792	2	0.673	5	−0.809	0.47
F_1_ʹ (R♀ × S♂)	0.139	0.078– 0.254	1.58 ± 0.37	0.915	2	0.632	4.48	−0.856	0.44
F_1_(Pooled)	0.146	0.116–0.184	1.52 ± 0.26	0.253	2	0.881	4.70	−0.835	
BC_1_((F_1_♂ × R♀)	0.268	0.127–0.412	0.84 ± 0.35	0.113	2	0.945	8.64	−0.571	
BC_2_(F_1_♀ × R♂)	0.347	0.218–0.528	1.28 ± 0.23	1.286	3	0.732	11.19	−459	

CL, the 95% confidence limits of LC_50_.

SE, standard error of the mean value.

χ^2^, Chi-square value.

Df, degree of freedom.

*P*, the probability value.

RR,(resistance ratio) = LC_50_ (Indox-SEL (R), F_1_, F_1_ʹ, BC_1_, or BC_2_)/LC_50_ Lab-Pop (S).

DLC, the degree of dominance=$D_{LC}=Log\;LC_{50}F1\text{-}Log\;LC_{50}\;SS/Log\;LC_{50}RR\text{-}LogLC_{50}SS$. (DLC) ranges from 0 (completely recessive) to 1(completely dominant) (Bourguet and Raymond 1998).

Log, the logarithms of LC_50_ values for the (*S*) and (*R*) strains and the reciprocal progeny F_1_ and F_1_ʹ and backcross BC_1_ and BC_2_.

### Dominance of Resistance

Based on LC_50_ values for F_1_ (R♂ × S♀) and F_1_ʹ (R♀ × S♂), the degree of dominance (*D*) recorded 0.47 and 0.44, respectively (Table 2). In addition, the results of effective dominance (*D*_ML_) showed that the extent of dominance of indoxacarb resistance ranged from incompletely dominant to completely recessive with the highest concentration (2 mgL^−1^) (Table 3). The monogenic inheritance model in the back cross showed a significant difference between the observed and expected mortalities at all concentrations of indoxacarb (*P* < 0.05), proving that resistance to indoxacarb in the Indoxa-SEL strain has a polygenic inheritance mode (Table 4).

**Table 3.** Effective dominance (*D*_*ML*_) of Indoxacarb resistance in *Spodoptera littoralis*

Concentration (ppm)	Population	Mortality (%)	Effective dominance (*D*_*ML*_)
2	Lab-Pop (S)	100	0.00 Incomplete recessive
	Indox-SEL (R)	80.00	
	F1	100	
1	Lab-Pop (S)	100	0.11 Incomplete recessive
	Indox-SEL (R)	53.33	
	F_1_	95.00	
0.5	Lab-Pop (S)	100	0.13 Incomplete recessive
	Indox-SEL (R)	23.33	
	F_1_	90.00	
0.25	Lab-Pop (S)	86.67	0.28 Incomplete recessive
	Indox-SEL (R)	10.00	
	F_1_	65.00	
0.125	Lab-Pop (S)	80.00	0.45 Incomplete recessive
	Indox-SEL (R)	3.00	
	F_1_	45.00	

Effective dominance (*D*_*ML*_) (completely recessive if D_ML_ = 0, but for a completely dominant resistance D_ML_ = 1). $D_{ML}=(MT_{RS}-MT_{SS})/(MT_{RR}-MT_{SS})$(Bourguet et al. 2000).

**Table 4.** Chi-squared analysis for monogenic or polygenic inheritance of indoxacarb resistance in *Spodoptera littoralis*

Concentration (ppm)	Number of larvae	Observed response (F)	Expected response (p)	*q*(1–*p*)	χ^2^(df = 1)	*P*
BC_1_(F_1_♀ × R♂)						
2	30	83.33	87.48	12.52	196.51	<0.00001
1	30	76.66	71.58	28.42	70.26	<0.00001
0.5	30	50	52.58	47.42	31.18	<0.00001
0.25	30	46.66	36.16	63.84	15.56	0.00008
0.062	30	16.,66	14.32	85.68	4.63	0.031418
					$\sum \chi^{2}$ = 318.14 df = 4	<0.00001
BC_2_((F_1_♂ × R♀)						
2	30	76.66	87.48	12.52	197.54	< 0.00001
1	30	70	71.58	28.42	70.71	< 0.00001
0.5	30	56.66	52.58	47.42	30.91	<0.00001
0.25	30	50	36.16	63.84	15.46	0.000084
					$\sum \chi^{2}$ = 314.62 df = 3	< 0.00001

^*^Significant difference (*P* < 0.05) between the observed and expected χ^2^ values.

### Number of Genes Involved

As shown in Table 4, the direct test of a monogenic model showed that at all the tested concentrations, significant differences were noticed between observed and expected mortality (*P* < 0.05).

### Realized Heritability (*h*^2^) and Projected Rate of Indoxacarb resistance

**Fig. 1.**
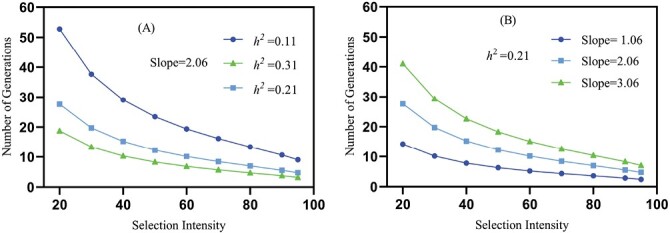
Effect of A) different heritability and B) different slopes on the number of generations of CLW required for a 10-fold increase in the LC_50_ of indoxacarb at different selection intensities.

Based on realized heritability calculation illustrated in Table 5, the projected rate of increase in indoxacarb resistance is directly proportional to selection intensity. Considering a mean slope of 2.06, 12.29 to 4.75 generations are required for a 10-fold rise in LC_50_ at 50–95% selection intensity if *h*^2^ = 0.21. However, 8.32 to 3.22 generations are required at the same slope and selection intensity if *h*^2^ = 0.31. On the other hand, it will take only 23.46 to 9.07 generations if *h*^2^ = 0.11 (Fig. 1A). Projected rate of resistance development is inversely proportional to slope. Oppositely, taking a constant value of heritability (*h*^2^ = 0.21 in the current study), with a slope of 1.06, then 6.32 to 2.44 generations will be needed for the 10-fold rise in LC_50_ at 50–95% selection intensity at each generation. When the slope was 3.06, 18.25 to 7.06 generations were required (Fig. 1B).

**Table 5.** Estimation of realized heritability (*h*^2^) of resistance to indoxacarb in laboratory-selected strain of *Spodoptera littoralis*(G0–G15)

Generations	*N*	Estimate of mean response per generation			Estimate of mean selection differential per generation				*h* ^2^
		Log of initial LC_50_	Log of final LC_50_	*R*	*I*	Mean slope	*σp*	S	
(G_0_–G_7_)	8	–1.40	–0.24	0.15	0.798	2.05	0.48	0.38	0.39
(G_7_–G_15_)	8	–0.24	–0.07	0.02	0.798	2.07	0.48	0.38	0.05
(G_0_–G_15_)	16	–1.40	–0.07	0.08	0.798	2.06	0.48	0.38	0.21

Initial and final LC_50_ values (µgml^−1^) were determined at G_0_, G_7_ and G_15,_ respectively.

R, response to selection: [log (final LC_50_) − log (initial LC_50_)]/*n*.

I, intensity of selection.

*σp*, phenotypic deviation [1/mean slope)].

S, selection differential (difference in mean phenotype between the selected parents and the entire parental generation, (*s* = *iσp*).

*h* ^2^, realized heritability (R/s).

### Fitness Comparison

The developmental duration of different stages for the Indoxa-S and Indoxa-SEL strain of *S. littoralis* is shown in Table 6. The development time of egg, pupae, preadult, and adult expanded significantly in the Indoxa-SEL strain compared with the susceptible one. Nevertheless, the larval stage showed a nonsignificant difference between both strains. Generally, the total longevity of the Indoxa-SEL strain exhibited a significant increase in the developmental duration. Likewise, the total preovipositional period of females (TPOP) for Indoxa-SEL strain showed a significant increase in the developmental duration. However, fecundity (egg/female) and adult preovipositional period of female adults showed a nonsignificant difference between both strains.

**Table 6.** Duration of the development, reproduction, survival rate, and life table parameters for the susceptible (Lab. Strain) and indoxacarb-resistant strains (Selected Strain) of *S. littoralis*

Stages	Lab. Strain	*N*	Selected strain	*N*	Statistical analysis	
	Mean ± SE		Mean ± SE		CI (95%) of difference	*P*
Egg (d)	5.49 ± 0.03	197	5.73 ± 0.03	189	(0.13,0.32)_ *_	0.0000
Larvae (d)	22.26 ± 0.13	164	22.33 ± 0.13	153	(–0.29,0.42)	0.7273
Pupae (d)	14.64 ± 0.15	164	15.39 ± 0.13	152	(0.34,1.15)_ *_	0.0002
Preadult (d)	42.41 ± 0.12	164	43.42 ± 0.11	152	(0.68,1.33)_ *_	0.0000
Adult (d)	12.78 ± 0.18	164	15.14 ± 0.15	152	(1.88,2.84) _*_	0.0000
Total longevity (d)	55.19 ± 0.22	198	58.56 ± 0.19	189	(2.77,3.96)_ *_	0.0000
Fecundity (egg/female)	1286.54 ± 71.70	90	1223.13 ± 123.28	72	(–215.49, 342.52)	0.6538
APOP (d) ^*c*^	3.54 ± 0.24	90	3.14 ± 0.28	63	(–0.33,1.13)	0.2803
TPOP (d) ^*d*^	45.38 ± 0.27	90	46.47 ± 0.34	63	(0.21,1.95)_ *_	0.0145
*O*_*d*_ (d)^*e*^	5.11 ± 0.18	90	5.82 ± 0.38	63	(–0.11,1.54)	0.0917

Means a significant difference at *P* < 0.05.

*N*, the number of individuals.

SE, standard error of the mean value.

CI, the paired bootstrap test on the difference between Lab. Strain and indoxacarb-resistant strain. If the CI includes 0, there is no difference at the 5% level.

d, days.

APOP, adult preovipositional period of female adult.

TPOP, total preovipositional period of female counted from birth.

*O*_*d*_, oviposition days.

**Fig. 2.**
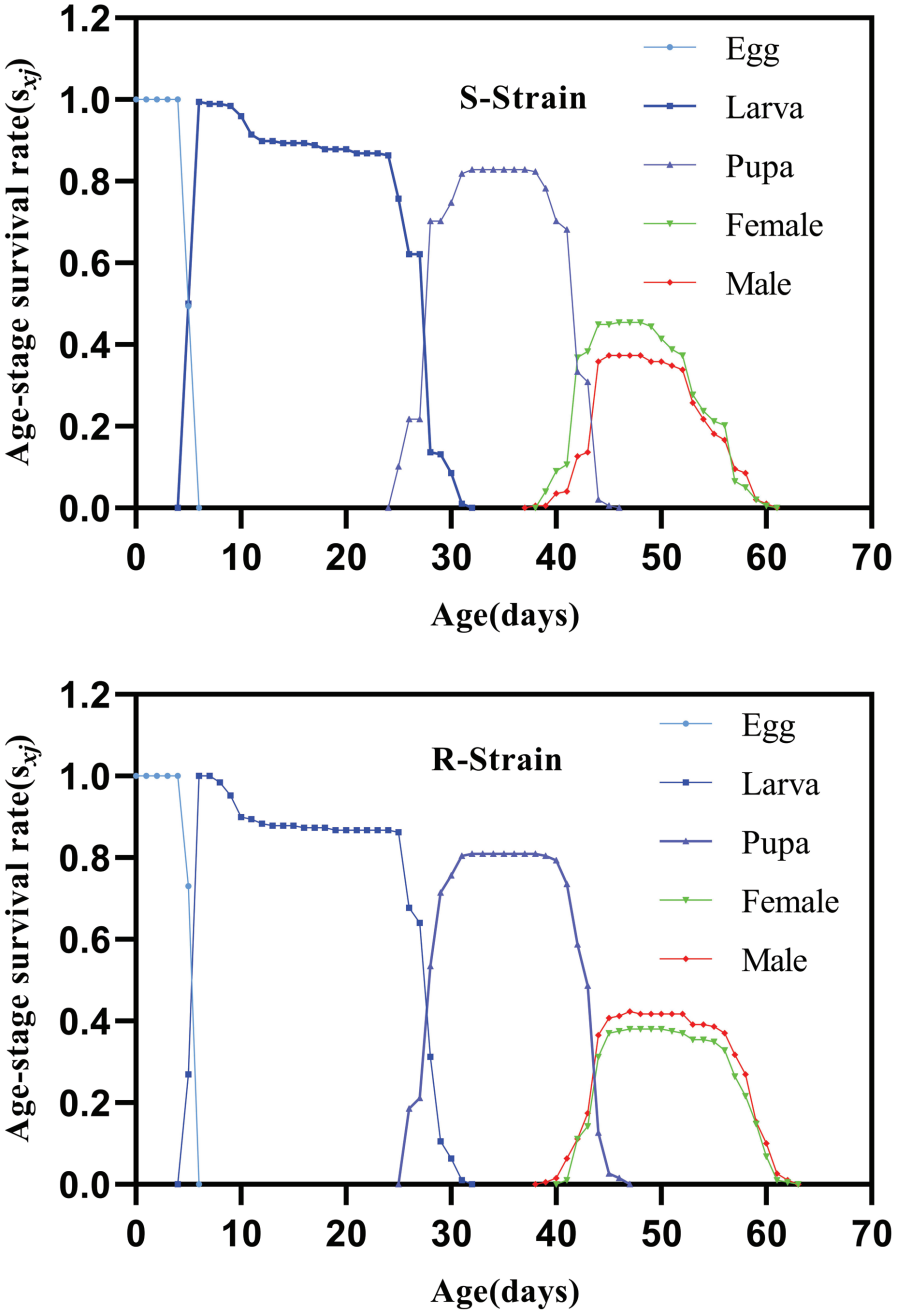
Age-stage-specific survival rates (*S*_*xj*_) of susceptible and resistant strains of *S. littoralis.*

**Fig. 3.**
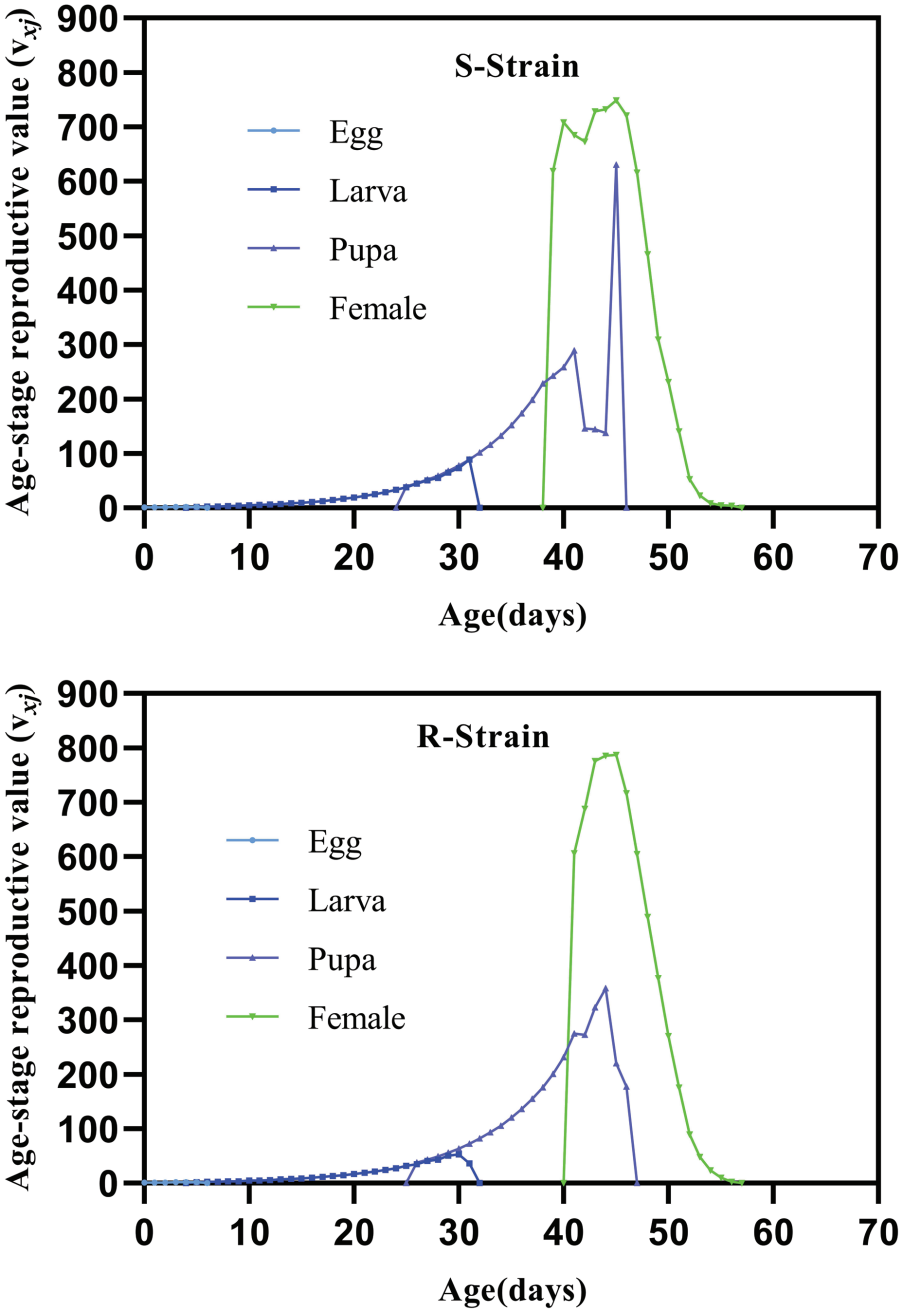
Age-stage reproductive value (*v*_*xj*_) of susceptible and resistant strains of *S. littoralis.*

**Fig. 4.**
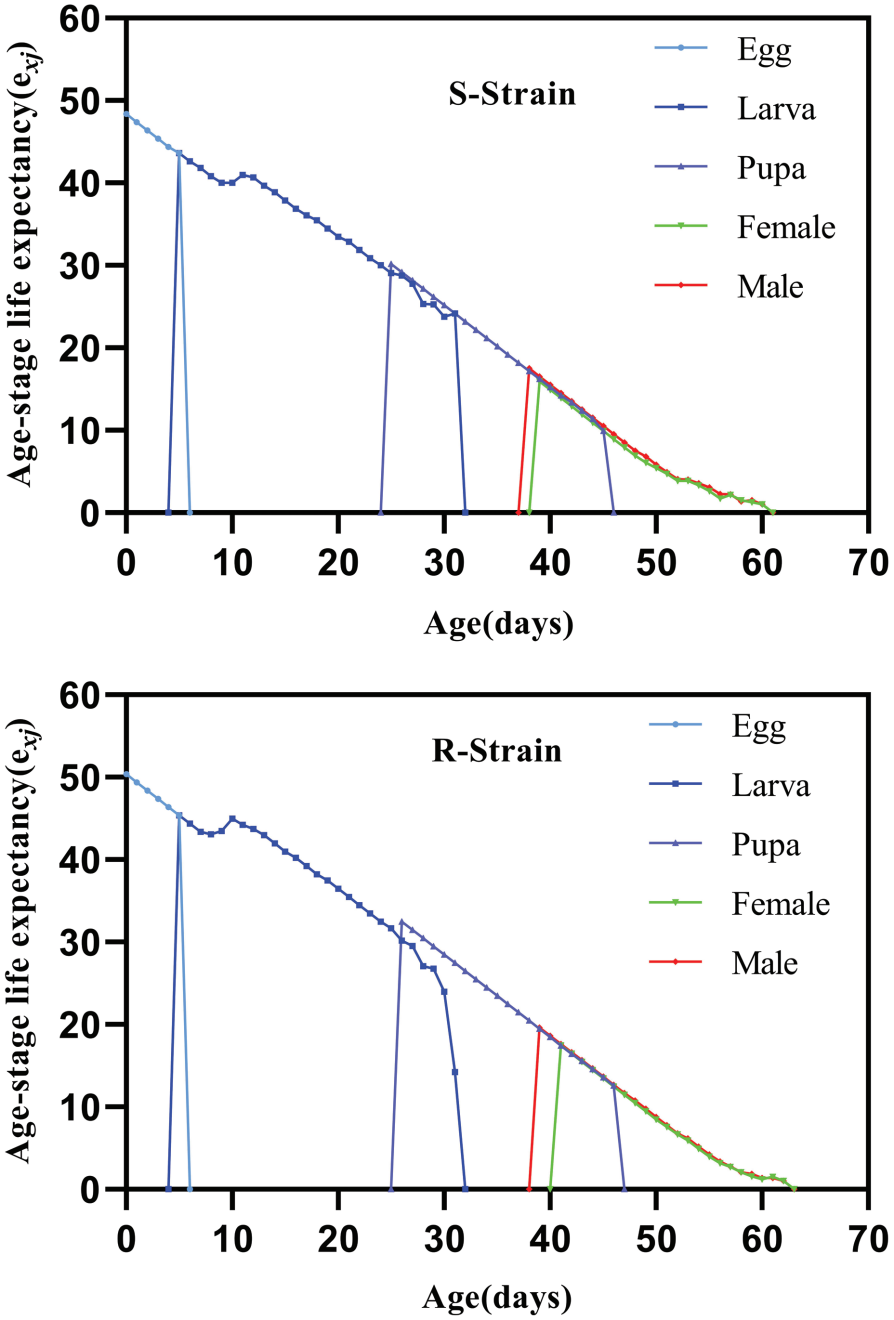
Age-stage-specific life expectancy (*e*_*xj*_) of susceptible and resistant strains of *S. littoralis.*

**Fig. 5.**
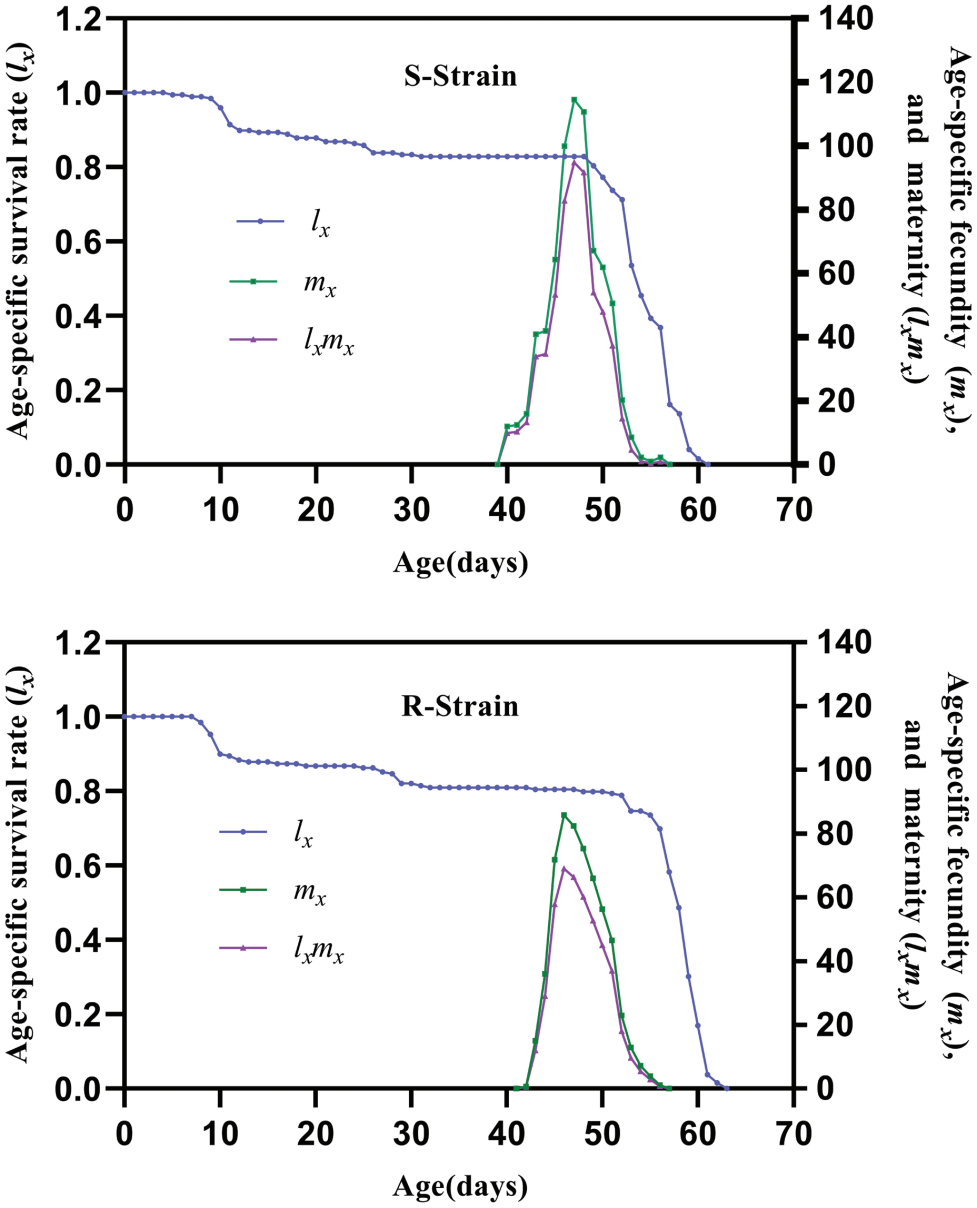
Age-specific survival rate (*l*_*x*_), age-specific fecundity of total population (*m*_*x*_), and age-specific maternity (*l*_*x*_*m*_*x*_) of susceptible and resistant strains of *S. littoralis.*

On the other hand, population growth parameters, including intrinsic rate of increase (*r*), finite rate of increase (*λ*), net reproductive rate (*R*_0_), doubling time (DT), and growth reproductive rate (GRR, were not significantly different between both strains. Nonetheless, the only parameter that was affected was the mean generation time (*T*) which showed a significant increase in Indoxa-SEL strain compared with the susceptible one. Consequently, compared with the susceptible strain, the calculated relative fitness of the Indoxa-SEL strain was 0.80 (Table 7). The survival-related curves including age-stage-specific survival rate (*S*_*xj*_), age-specific survival rate (*l*_*x*_), and age-specific maternity (*l*_*x*_*m*_*x*_) showed a similar trend among the Indoxa-SEL and Indoxa-S strain (Figs. 2 and 5). Likewise, graphs for age-stage reproductive value (*v*_*xj*_) and age-stage-specific life expectancy (*e*_*xj*_) showed obvious similarity between both strains (Figs. 3 and 4). In addition, the graph of age-specific fecundity (*m*_*x*_) indicated that no significant difference in fecundity levels was observed between Indoxa-SEL strain and Indoxa-S strain (Fig. 5).

**Table 7.** Population parameters of the susceptible (Lab. Strain) and indoxacarb-resistant strains (Selected Strain) of *S. littoralis*

Parameters	Lab. Strain (Mean ± SE)	Selected Strain (Mean ± SE)	Statistical analysis	
			95% CI of difference	*P*
GRR	726.41 ± 65.84	583.66 ± 77.41	(–126.67,269.11)	0.79134
Net reproductive rate (*R*_0_) (offspring/individual)	584.79 ± 55.86	465.95 ± 63.94	(–105.79,224.42)	0.79136
intrinsic rate of increase (*r*) (*d*^−1^)	0.1342 ± 0.0022	0.1271 ± 0.0030	(–0.0051, 0.0123)	0.75787
Finite rate of increase (*λ*) (*d*^−1^)	1.1436 ± 0.0025	1.1355 ± 0.0033	(–0.00005,0.0164)	0.05181
Mean generation time *T* (*d*)	47.46 ± 0.28	48.33 ± 0.24	(0.14,1.59) _*_	0.0199
The doubling time (DT)	5.16 ± 0.09	5.45 ± 0.13	(–0.008,0.59)	0.05742
Rf (Relative fitness)	1	0.80		

Means a significant difference at *P* < 0.05.

SE, standard error of the mean value.

d, days.

CI, the paired bootstrap test on the difference between Lab. Strain and indoxacarb-resistant strain. If the CI includes 0, there is no difference at the 5% level.

Relative fitness (Rf) = *R*_0_ of indoxacarb selected strain/*R*_0_ of the Lab. Strain.

## Discussion

Indoxacarb, a sodium channel blocker insecticide, plays a fundamental role in pest control. Due to the intensive use, resistance to indoxacarb had been reported in field populations of *Plutella xylostella* (Wang et al. 2016, Zhang et al. 2016), *Helicoverpa armigera* (Qayyum et al. 2015), *Spodoptera exigua* (Gao et al. 2014), and *Spodoptera litura* (Shi et al. 2022). In the current study, laboratory selection of a field population of *S. littoralis* slowly developed resistance to indoxacarb. Numerous insect species have reported similar patterns such as *P. xylostella*, which exhibited 31.3-fold resistance after selection for 10 generations (Nehare et al. 2010) and 85.4-fold resistance after selection for 53 generations (Zhang et al. 2017) and *H. armigera* which showed 4.43-fold after 11 selected generations (Cui et al. 2018). In contrast, high resistance levels to indoxacarb have been reported in *Spodoptera exigua* (240-fold after 12 generations) (Gao et al. 2014), *Helicoverpa armigera* (1,239-fold after 8 generations) (Ghodki et al. 2009), *Spodoptera litura* (95-fold after 3 generations) (Sayyed et al. 2008), and *Spodoptera frugiperda* (299.45-fold after 24 generations). These disparities may be due to differences in species’ geographical origin or to the effects of initial sampling (Hafeez et al. 2022).

In the current study, the inheritance pattern of indoxacarb resistance in *S*. *littoralis* was investigated by using reciprocal and back-cross experiments. The obtained data revealed that resistance to indoxacarb was autosomal, incompletely recessive, and polygenic. The toxicity data of reciprocal crosses F_1_ and F_1_ʹ suggested that the inheritance of indoxacarb resistance in *S*. *littoralis* was autosomal as there was no significant difference in the LC_50_ values of both reciprocal crosses. Similarly, autosomal nature of indoxacarb resistance was reported in *S. frugiperda* (Hafeez et al. 2022), the diamondback moth, *Plutella xylostella* (L.) (Marak et al. 2017), *Helicoverpa armigera* (Hubner) (Ghodki et al. 2009), *S. litura* (Lepidoptera: Noctuidae) (Sayyed et al. 2008), and *Phenacoccus solenopsis* Tinsley (Homoptera: Pseudococcidae) (Afzal and Shad 2016).

In this study, resistance dominance to indoxacarb in *S. littoralis* was evaluated through 2 methods: degree of dominance (*D*_LC_) and *D*_*ML*_. The degree of dominance of F1 and F1ʹ were 0.47 and 0.44, respectively indicating that indoxacarb resistance in *S. littoralis* was incompletely recessive. The similarity between the degree of dominance for F_1_ and F_1_ʹ, which is recorded for *S. littoralis* in our study and in the previously mentioned insects, interpreted indoxacarb resistance as a common degradation mechanism (Hafez et al. 2020). An incompletely recessive mode of inheritance of indoxacarb resistance was also observed in *H. armigera* (Cui et al. 2018), and *Plutella xylostella* (L.) (Sayyed and Wright 2006, Nehare et al. 2010). In contrast, incompletely dominant indoxacarb resistance was observed in *S. litura* (Sayyed et al. 2008), *Phenacoccus solenopsis* Tinsley (Homoptera: Pseudococcidae) (Afzal and Shad 2016), and *Helicoverpa armigera* (Lepidoptera: Noctuidae) (Bird 2017).

Further genetic analysis showed that the *D*_*ML*_ of resistance to indoxacarb depended upon the tested concentration of the insecticide. In our study, the *D*_*ML*_ value for each of the tested 5 concentrations showed that dominance increased as indoxacarb concentration increased. These results suggest that dominance level is not a fixed parameter and that it varies according to the insecticide concentration. Consistent with our results, the pattern of concentration-dependent dominance of indoxacarb was reported in various studies (Sayyed and Wright 2006, Sayyed et al. 2008, Afzal and Shad 2016, Bird 2017).

Insecticide resistance to chemical insecticides can be either monogenic (single locus with major effect) or polygenic (multiple loci with additive effect) (Roush 1998, Ffrench-Constant et al. 2004). Polygenic resistance is most common under laboratory continuous selection due to the absence of rare variants in the laboratory (McKenzie et al. 1992, Abbas et al. 2014). Generally, the resistance controlled by multiple genes evolves more slowly than that controlled by a single gene. In addition, it is usually more difficult to manage, and can spread faster with dominant genes (Barnes et al. 1995, Lira et al. 2020).

In the present work, the monogenic model, based upon *χ*^2^ goodness of fit tests, showed significant deviations (*P* < 0.05) between observed and expected mortality for all tested concentrations, suggesting that indoxacarb resistance in S. *littoralis* is controlled by multiple loci. In conformity with this, the polygenic nature of indoxacarb resistance has been reported in *S. litura* (Sayyed et al. 2008), *Phenacoccus solenopsis* Tinsley (Homoptera: Pseudococcidae) (Afzal and Shad 2016). Nevertheless, indoxacarb resistance was reported to be monogenic in *Helicoverpa armigera* (Lepidoptera: Noctuidae) (Bird 2017), the diamondback moth, *Plutella xylostella* L. (Lepidoptera: Plutellidae) (Sayyed and Wright 2006), and *Spodoptera exigua* (Hübner) (Wang et al. 2011). The variation in insecticides resistance mode of inheritance may be attributed to dissimilar selection history, insecticidal types, and genetic backgrounds (Roush and McKenzie, 1987).

Resistance risk assessment contributes to maintaining the effectiveness of the used insecticides, delaying resistance resurgence, and enhancing insecticide integration into resistance management programs (Tabashnik 1992). Realized heritability (*h*^2^) is an effective tool used to detect the potential for resistance development by using a brief selection experiment (4–6 generations). Realized heritability (*h*^2^) value represents the heritability of resistance to a certain stressor (insecticide) and clarifies how a population responds to selection by this stressor (Klerks et al. 2011).

In the present study, the *h*^2^ of indoxacarb resistance in *S. littoralis* was 0.21 in the entire selection process, which is lower than that obtained in the first half (G0–G7) of selection (*h*^2^ = 0.39), and higher than that observed in the second half (G7–G15) (*h*^2^ = 0.05). Additionally, the selection response (*R*) declined while the selection differential (*S*) was stable as the indoxacarb selection progressed, leading to higher *h*^2^ in the first half than in the second half of selection. These results indicate that the additive genetic variation of indoxacarb resistance in *S. littoralis* was present initially, and then declined in the second round of selection. It is reported that the estimated realized heritability values (*h*^2^) of indoxacarb resistance recorded different values with different pests. *h*^2^ values recorded 0.37 in *S. littoralis* (Mokbel et al. 2017), 0.07 in *Helicoverpa armigera* (Hübner) (Lepidoptera: Noctuidae) (Cui et al. 2018), 0.12 in *Spodoptera litura* (lepidoptera: Noctuidae) (Sayyed et al. 2008), 0.04 in *Phenacoccus solenopsis* (Homoptera: Pseudococcidae) (Afzal and Shad 2016). The different *h*^2^ values in different insect pests indicate different potential to develop resistance to indoxacarb due to the different frequencies of resistant alleles, and consequently, genetic variations.

Insecticide resistance evolution is often accompanied by a decline in biological fitness which in turn limits the development of insect resistance (Sial et al. 2011). Reduction of the insect’s biological fitness may be due to the requirement for a high energetic cost. Therefore, investigating the fitness costs of insecticide resistance is necessary for designing potential resistance management strategies (Kliot and Ghanim 2012). To verify the fitness costs of indoxacarb-resistant in *S. littoralis* in this study, we compared the fitness components of indoxacarb-resistant and susceptible strains. Our results indicated that the indoxacarb-resistant strain showed a significant increase in the developmental duration of the egg, pupae, preadult, adult, total longevity, and TPOP compared to the susceptible strain. These effects have also been reported in the fall armyworm, *Spodoptera frugiperda* (J.E. Smith) (Hafeez et al. 2022) and *Phenacoccus solenopsis* Tinsley (Homoptera: Pseudococcidae) (Afzal et al. 2015). Concerning life table parameters, the obtained results showed no major cost associated with the net reproductive rate (*R*_0_), intrinsic rate of increase (*r*), finite rate of increase (*λ*), and the DT in indoxacarb-resistant strain of *S. littoralis*. Consistently, indoxacarb-resistant *Helicoverpa armigera* (Lepidoptera: Noctuidae) did not confer a major fitness cost compared with the susceptible strains (Bird et al. 2020). The obtained data exhibited a lack of fitness costs related to indoxacarb resistance with a relative fitness value of 0.80, compared with susceptible strain.

To summarize, in this study, we reported the characterization of *S. littoralis* resistance to indoxacarb. *S. littoralis* resistance to indoxacarb was found to be autosomal, incompletely recessive, and polygenic. Realized heritability estimation proved the potential of *S. littoralis* to develop resistance to indoxacarb. Furthermore, our work forecast the rate of resistance development under various selection intensities and populations homozygous conditions. The current work will contribute to conserving indoxacarb efficacy and designing effective resistance management programs against *S. littoralis*.
